# Advances in Plant Auxin Biology: Synthesis, Metabolism, Signaling, Interaction with Other Hormones, and Roles under Abiotic Stress

**DOI:** 10.3390/plants13172523

**Published:** 2024-09-08

**Authors:** Jianshuang Gao, Shunyao Zhuang, Weiwei Zhang

**Affiliations:** 1 State Key Lab of Soil and Sustainable Agriculture, Institute of Soil Science, Chinese Academy of Sciences, Nanjing 210008, China; gaojianshuang@hufe.edu.cn (J.G.); zhangweiwei@issas.ac.cn (W.Z.); 2 School of Economic Geography, Hunan University of Finance and Economics, Changsha 410205, China

**Keywords:** auxin, auxin signal transduction, abiotic stress, hormone

## Abstract

Auxin is a key hormone that regulates plant growth and development, including plant shape and sensitivity to environmental changes. Auxin is biosynthesized and metabolized via many parallel pathways, and it is sensed and transduced by both normal and atypical pathways. The production, catabolism, and signal transduction pathways of auxin primarily govern its role in plant growth and development, and in the response to stress. Recent research has discovered that auxin not only responds to intrinsic developmental signals, but also mediates various environmental signals (e.g., drought, heavy metals, and temperature stresses) and interacts with hormones such as cytokinin, abscisic acid, gibberellin, and ethylene, all of which are involved in the regulation of plant growth and development, as well as the maintenance of homeostatic equilibrium in plant cells. In this review, we discuss the latest research on auxin types, biosynthesis and metabolism, polar transport, signaling pathways, and interactions with other hormones. We also summarize the important role of auxin in plants under abiotic stresses. These discussions provide new perspectives to understand the molecular mechanisms of auxin’s functions in plant development.

## 1. Introduction

**Figure 1:**
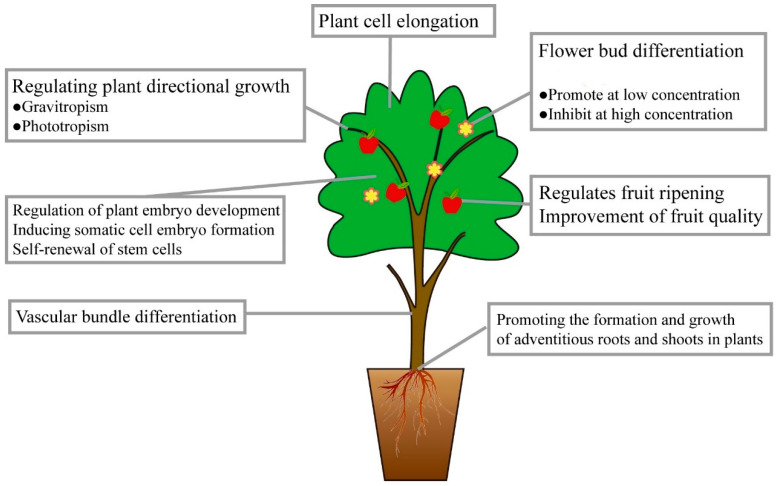
Schematic presentation of auxin function in plants.

The hormone auxin, which is composed of various substances with growth-inducing effects, is important in plant physiology [1]. Auxin is an endogenous hormone characterized by the presence of an unsaturated aromatic ring and an acetic acid side chain [2,3]. It was first discovered in coleoptile experiments by Darwin [4]. In addition, auxin refers to a class of small molecule compounds that primarily act through polar transport and signal transduction, and auxin production and metabolism influence how plants grow, develop, and respond to external stimuli to adapt to their changing environment [5]. The establishment and maintenance of polarity, apical dominance, phototropism, gravity, senescence, pathogen response, abiotic stress responses, and fruit formation are only a few of the processes controlled by auxin during plant growth and development [6] (Figure 1). Auxins have been used widely in the field of agricultural production, greatly improving the yield and quality of crops, and creating significant social and economic benefits.

The effects of auxins on plant development have been widely studied. It has been found that auxins play a key role in regulating the formation of adventitious roots, forms, types, and concentrations of auxins also have different impacts on this process [7,8]. In particular, the level of free auxins in plant tissues is crucial for growth and development of plants. *Arabidopsis thaliana* can form adventitious roots from young leaf explant without exogenous auxins, whereas exogenous auxins induce older leaf explants to form adventitious roots [9]. Auxins are often used to induce parthenocarpy, increase fruit set, and inhibit flowering, as well as to improve low fruit maturation rates [10,11]. Moreover, studies have shown that indole-3-acetic acid (IAA) affects plant flowering, but the specific mechanism is still unclear. Most studies suggest that low concentrations of IAA promote flowering, while high concentrations inhibit it [12,13]. The development of neighboring organs is inhibited or retarded by a floral organ that produces high levels of free auxin. During floral bud development, young organs that produce high levels of free IAA inhibit or delay the initiation and development of organ primordia at the shoot tip [12]. Zhao et al. found that higher IAA level resulted in longer hypocotyls and shorter primary roots in *yucca*, an *Arabidopsis* activation-tagged mutant [14]. On the other hand, results of metabolic and transcriptomic analyses indicate that auxin participants in the early stages of fruit development, and a corresponding increase in sugar at the ripening stages [15,16]. Furthermore, the maturity of strawberries was negatively correlated with the IAA content [17,18].

Auxins not only respond to intrinsic developmental signals, but also mediate various environmental signals, participating in the regulation of plant growth and development and growth responses, such as gravity and light signals. For example, the asymmetric distribution of auxins is essential for the formation of plant gravitropic responses, which is mainly achieved by auxin polar transport and signal transduction [19,20,21,22,23]. The asymmetric distribution of auxins is also the main cause of plant phototropic growth, which is mainly regulated by various auxin transporters [24,25,26,27,28,29,30,31].

Auxin plays an important role in regulating plant responses to various stressors and has received much research attention in recent years. Abiotic stressors, such as drought, salinity, and low temperature, have imposed increasingly serious constraints on the stable and high yield of grains. Since the beginning of this century, global grain production has been mainly affected by abiotic stresses [32]. Early in the vegetative growth stage, abiotic stress impacts the development and differentiation of plant cells. Abiotic stress during the reproductive growth stage might cause a large reduction in yield or possibly no harvest at all [33]. Auxin is one of the hormones that plants deploy during stress to maintain their homeostasis in vivo and lessen the negative effects of stress on plant growth and development [34,35]. 

Auxin affects the yield, quality, and resistance of plants by regulating the important signal molecules required for their plastic growth and development, especially of roots. This paper reviews research on indole-acetic acid (IAA, an auxin) synthesis and metabolism, polar transport, and signal transduction in recent years to provide a reference for the use of IAA in high-quality cultivation and rapid plant propagation.

## 2. Auxin Types

**Figure 2:**
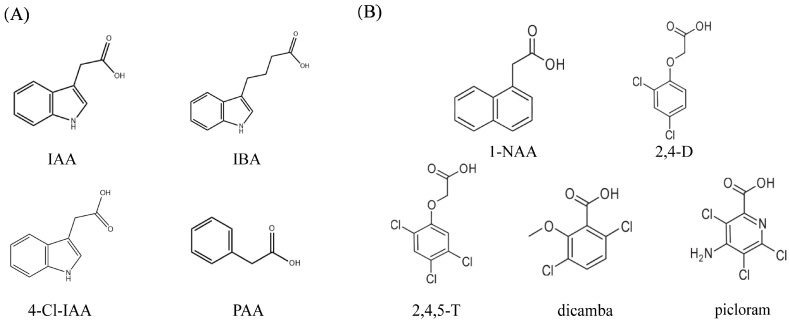
Examples of endogenous auxins (**A**) and some synthetic auxins (**B**) are presented. (**A**) IAA: indole-acetic acid; IBA: indole-3-butyric acid; 4-Cl-IAA: 4-chloroindole-3-acetic acid; and PAA: phenyl-acetic acid. (**B**) 1-NAA: 1-Naphthalene-acetic acid; 2,4-D: 2,4-dichlorophenoxyacetic acid; 2,4,5-T: 2,4,5-trichlorophenoxy-acetic acid; dicamba: 3,6-dichloro-2-methoxybenzoic acid, and picloram: 4-Amino-3,5,6-trichloropicolinic acid [48].

Currently, there are two main categories of known auxins: endogenous auxins and synthetic auxins (Figure 2). Endogenous auxins mainly include IAA, indole-3-butyric acid (IBA), 4-chloro-indole-3-acetic acid (4-C1-IAA), and phenylacetic acid (PAA). They exist in two forms, bound and in a free state [36,37,38,39,40]. Initially, IBA was discovered in the tuber of the horse bell, but has since been discovered in other plants. According to Campanella et al. (2004), IBA accounts for 25–30% of total Arabidopsis auxin [41]. This auxin is widely used in agricultural production as a rooting agent, and also participates in auxin-mediated leaf formation, cell division, stem bending, and root hair formation [42,43]. Originally, 4-C1-IAA was found in immature pea seeds; however, the model plant *Arabidopsis thaliana* does not produce this form of auxin. The main roles of 4-C1-IAA are the promotion of pea seed coat development and the elongation of corn colloblasts [44]. Phenylacetic acid is the only phenyl-derived endogenous auxin found so far, which is mainly involved in the interaction between roots and soil microorganisms [45]. Auxin used as a plant growth regulator mostly consists of synthetic auxins, such as NAA, 2, 4-D, trichlorophenoxyacetic acid (2, 4, 5-T), and picloram, among others [46]. These synthetic auxins are more stable than IAA [47].

## 3. Auxin Synthesis

**Figure 3:**
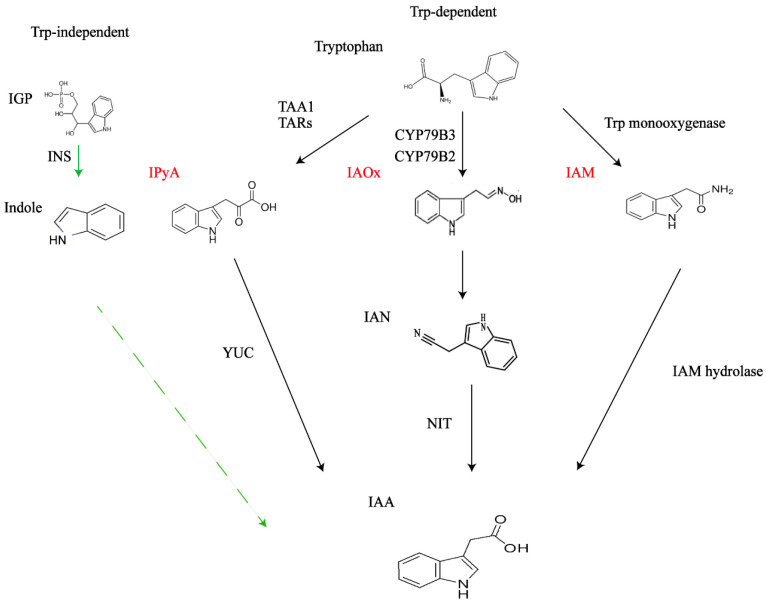
A model of the tryptophan (Trp)-dependent and Trp-independent indole acetic acid (IAA) biosynthetic pathways. IGP, indole-3-glycerol phosphate; INS, indole synthase gene; TAA1, tryptophan aminotransferase; TARs, TAA1-associated proteins; IPyA, indole-3-pyruvate; YUC, YUCCA; IAOx, indole-3-acetaldoxime; IAM, indole-3-acetamide; CYP79B2 and CYP79B3 in the cellular phosphorus P450 (CYP) mono-oxygenase family.

Auxin is synthesized in plants via several pathways [49]. The metabolism of IAA mainly can be divided into two kinds synthesis pathways: tryptophan (Trp)-dependent and tryptophan-independent [50,51] (Figure 3). Wang et al. (2020) suggested that the cytoplasmic enzyme indole synthase (INS) may be a key enzyme in the Trp-independent IAA biosynthetic pathway [52]. However, little is known about the molecular components and physiological functions of the Trp-independent pathway. The tryptophan-dependent auxin synthesis pathway dominates in plants, and IAA synthesis pathways are classified as the indole-3-acetaldoxime (IAOx) pathway, the indole-3-acetamide (IAM) pathway, and the indole-3-pyruvate (IPyA) pathway [53] (Figure 3). The indole synthase gene (*INS*) is the primary gene in the tryptophan-independent auxin synthesis pathway [54]. Indole, also known as indole glycerophospholipid (IGP), is a key node in both the tryptophan-dependent and tryptophan-independent pathways of auxin production [55]. The following focuses on the tryptophan-dependent auxin synthesis pathway. It is mainly mediated by transaminases and decarboxylases [56].

While other redundant processes function in parallel, the IPyA path is a significant and often conserved mechanism for IAA production in plants. It mainly involves two reactions: First, tryptophan is deaminated to IPyA by tryptophan aminotransferase (TAA1) and TAA1-associated proteins (TARs) in Arabidopsis [57,58]. IPyA is then decarboxylated to IAA by an irreversible reaction catalyzed by flavin-containing monooxygenases of the YUCCA (YUC) family. Trp is first converted to IPA by Trp transaminase and subsequently catalyzed by YUCase to produce IAA [2,59]. Studies have shown that ATA1 and YUC co-originals have been found in the genome of plants [60,61,62]. In *Marchantia polymorpha*, knockout of a single TAA gene results in loss of cell and tissue differentiation leading to severe growth and developmental defects [62]. Therefore, IPyA pathways are the main pathways of IAA biosynthesis in plants. 

The IAOx and IAM pathways play only minor roles in IAA homeostasis. IAOx, IAM, and IAN (indole-3-acetonitrile) are intermediates in the biosynthesis of IAA [63]. The conversion of amino acids to IAOx is mediated by the two related enzymes CYP79B2 and CYPs79B3 in the cellular phosphorus P450 (CYP) mono-oxygenase family [64,65]. IAOx is a precursor of indole glucosides (IGs) and camalexin, which act as defense metabolites in plants [66,67]. So far, both IAOx and CYP79B2/3 genes have been found only in Brassica [63], suggesting that this pathway is restricted to Brassicaceae. IAOx is synthesized from Trp catalyzed by the enzyme CYP79B2/B3, converted directly to IAN, and later generates IAA in the presence of nitrilase (NIT) [68]. Tryptophan is converted to IAA by the formation of IAM. The pathway begins with the conversion of tryptophan to IAM and IAM hydrolysis products catalyzed by the Trp monooxygenase, the former in the production of auxin in the presence of IAM hydrolase. The latter is directly mediated by indole precursors [69]. In Arabidopsis, disruption of the major IAM hydrolases IAMH1 and IAMH2 did not result in substantial developmental defects or changes in IAA content. It is suggested that the IAM pathway plays only a secondary role in growth hormone homeostasis [69].

## 4. Auxin Metabolism

The metabolism of IAA mainly proceeds the following three ways: (1) The formation of auxin conjugates, such as amide conjugates with amino acids and polypeptides, and the formation of ester conjugates with polysaccharides and inositol, which are generally used for the transport and storage of auxin [40,70,71]. (2) Conversion to IBA, which is more stable than IAA and can produce a variety of conjugates [72]. (3) Oxidative decomposition, in which IAA can be decomposed by oxidation of its side chain (decarboxylated) or the indole ring (non-decarboxylated). The decarboxylated oxidation process is more complex, and conjugated IAA is generally decomposed via non-decarboxylated oxidation [73]. This reaction is the oxidation of IAA to 2-oxoindole-3-acetic acid (oxaa), which is then glycosylated to oxaa-glc [74,75].

The predominant metabolic pathway for IAA is oxidative catabolism, as it was shown that oxaa is the most abundant IAA metabolite in *Arabidopsis* [76]. In algae, vascular and non-vascular land plants, oxidative catabolites are present at higher levels than amide-linked catabolic metabolites under normal physiological conditions. It suggests that oxidation is the major pathway for plant IAA catabolism [74,77,78,79]. IAA oxidase 1 (DIOXYGENASE FOR AUXIN OXIDATION 1, DAO1) is a member of the 2-oxoglutarate and iron (II)-dependent oxygenases superfamily [78]. Both Arabidopsis *AtDAO1* and rice *OsDAO* convert IAA to oxaa in vitro [80,81]. The *dao1-1* mutant exhibited an auxin accumulation phenotype, but plants overexpressing *atdao1* did not exhibit a significant auxin deficiency phenotype [78,81]. Loss of DAO1 function results in only minor developmental defects [80,82]. On the other hand, the GH3 gene encodes an acylamide synthase that catalyzes the coupling reactions of salicylic acid (SA), jasmonic acid (JA), and IAA with amino acids [83]. It was shown that the major natural auxin, IAA, is inactivated mainly through the GH3-ILR1DAO pathway [76]. First, IAA is converted to IAA-amino acid conjugates (IAA-aspartate (IAA-Asp) and IAA-glutamic acid (IAA-Glu) by GH3-type IAA amide synthase. DAO1 dioxygenase irreversibly oxidizes IAA—Asp and IAA—Glu to indole diketone-3 -acetic acid-aspartic acid (oxIAA-Asp) and oxIAA-Glu. oxIAA-Glu is then hydrolyzed by ILR1 to release inactive oxIAA [76,78]. It has been shown that DAO and GH3 enzymes play redundant roles in regulating IAA levels [78,81,84]. 

## 5. Auxin Transportation

Auxin can be transferred in higher plants in two ways: long-distance vascular transport and short-distance active transport requiring transport vehicles [85,86,87,88]. The latter is important in the asymmetric distribution of auxin, which is also known as auxin polar transport [89]. Three transport proteins are required for polar auxin transport (PAT): auxin-influx carrier AUXIN/LIKE-AUX (AUX1/LAX) family proteins, auxin-efflux carriers PIN-FORMED (PIN) family proteins, ATP-binding cassette B (ABCB) family proteins (Table 1). They are the main family of transporter proteins involved in PAT. Their quantity, polarity, and capacity to transport auxin at the PM influence the pace and directionality of intercellular auxin flow, establishing the pattern of auxin distribution [90,91]. These protein families are frequently functioned in plants to modulate auxin polar transport and distribution [92,93,94,95].

The AUX1/LAX family contains four highly homologous genes (*AUX1*, *LAX1*, *LAX2*, and *LAX3*) that encode transmembrane proteins in *Arabidopsis* [95,96]. The AUX1/LAX family is involved in a number of developmental processes, including embryogenesis, seed germination, leaf morphogenesis, vascularization, and root and terminal hook development [97,98]. The amount and polarity of the AUX1/LAX protein at the plasma membrane (PM) are strictly controlled. It helps to coordinate the distribution of growth factors essential for normal plant growth and development [99,100,101]. For example, in roots, asymmetric localization of AUX1 at the apical PM of protodermal cells promotes auxin flow toward the tip (root direction). While AUX1 is positioned at the base of the side roots and epidermal cells, it drives the flux to the base end (in the direction of the stem) [21]. In root columella cells, the increase in cytoplasmic AUX1 content implies a dynamic regulation of PM targeting and AUX1. Rapid subcellular localization and polarity regulation of AUX1 in root tissues can control auxin flow, which in turn regulates root growth in response to gravitational stimuli or other environmental inputs [21]. 

Two different transporters mediate growth hormone efflux, the ABC and PINs transporters. The ABCB family are nonpolar transporter proteins that are uniformly distributed along the PM [102,103]. Previously, ABCB1, ABCB4, and ABCB19 were considered to be nonpolar. However, it has been suggested that some homologs, including ABCB14 and ABCB15, may have polar membrane localization functions that contribute to the directionality of auxin flow [104,105]. On the other hand, polar-localized transport proteins (PINs) are components of the PAT machinery and have an important influence on the directionality of auxin flow in plant tissues and organs [106,107]. The eight members of the PIN family are transmembrane proteins, PIN1, PIN2, PIN3, PIN4, and PIN7 are localized to the PM, PIN5 and PIN8 are localized to the ER, and PIN6 is localized to the endoplasmic reticulum (ER) and PM [108]. The PIN located in the PM usually contains a long hydrophilic ring that separates multiple transmembrane structural domains, whereas the PIN located in the ER is characterized by a short hydrophilic ring in plants. And it has been shown that PIN transports auxin in unicellular plants [109,110], or in heterologous systems, including mammalian cells or Xenopus oocytes [109,111]. PINs primarily regulate physiology and development, such as embryogenesis, initiation, localization and formation of new organs, and tropic responses [107,112,113]. Interestingly, PINs and ABCB interact and control PAT in plants independently or interdependently [92,94].

**Table 1:** Types, coding genes, and functions of auxin transport proteins.

Types	Coding Genes	Functions	References
auxin-influx carrier	*AUX1*	*AUX1*: amino acids transporters and auxin permease activity; adventitious root development	[114,115,116,117,118,119,120,121,122,123]
	*LAX1*	*LAX1*: shoot and root pole formation,	
	*LAX2*	*LAX2*: xylem development, gravitropistic response, auxin distribution	
	*LAX3*	*LAX3*: auxin distribution, lateral root development, hook formation	
auxin-efflux carrier	*PIN (PIN-FORMED)* gene family,	*PIN1:* downward auxin transport, organ initiation, flower organ formation, leaf vein formation, and stem gravity response; *PIN2:* transport auxin from apex to elongation zone; root gravitropism *PIN3:* mediate auxin flow toward the lower hypocotyl side *PIN4* and *PIN7:* Auxin distribution during plant embryonic development *PIN5:* Transport auxin to the endoplasmic reticulum cavity *PIN6* and *PIN8:* Auxin transport across the plasma membrane	[23,104,106,113,124,125,126,127,128,129,130]
	*ABCB* gene family	*ABCB4, 14, 15, 19* and *21:* Auxin transport functions	[95,104,131]

## 6. Gravitropism and Phototropism of Auxin

Studies on the effects of auxin on gravitropism and phototropism have been investigated in recent years. A functionally deficient mutant of *AUXIN1* (*AUX1*), an auxin-influx carrier, with reduced gravity response when expressed in lateral root cap and epidermal cells [21]. The AtAUX1 protein acts together with the growth hormone export protein AtPIN2 to regulate the gravitropic response of the root system according to environmental signals and stimulation [20]. In the root columella, PIN3 is rapidly repositioned laterally in response to gravitational stimulation [22]. Plants exhibit gravitropic growth after perceiving gravity signals, and this process can be timely terminated to avoid excessive bending of plant tissues. For example, experiments on the mechanics of hypocotyl bending in *Arabidopsis thaliana* showed that when the hypocotyl stimulated by gravity for a short period of time (2–3 h), it began to show obvious gravitational response, which gradually weakened with the increase in processing time and almost disappeared after 30 h of processing [23]. Rakusova et al. (2016) found that this is due to an essential mechanism for restoring symmetry to PIN3-dependent auxin flow. PIN3 regulates gravity-mediated growth hormone transfer to the lateral hypocotyl and promotes its development. Afterwards PIN3 polarizes to the other side of the cell, accelerating auxin consumption to terminate the bend. However, pharmacological or genetic alterations prevent the PIN3 response from terminating, resulting in hyperbolic hypocotyls [23]. PIN3 and *PIN-FORMED7* (*PIN7*) modulate the directional transport and distribution of auxins on both sides of the root, further affecting the root response angle to gravity, and thus participate in the formation of root gravitropic morphology [19]. The asymmetric distribution of auxins is also the main cause of plant phototropic growth, which is mainly regulated by various auxin transporters. Plant hypocotyl phototropic bending is mainly regulated by auxin polar transport genes *PIN3*, *PIN4,* and *PIN7* [25]. ABCB-mediated (ATP-binding cassette B) auxin polar transport is involved in the regulation of hypocotyl growth by light signals, the regulation of which depends on light signal receptors *cryptochrome1* (*CRY1*), *phototropin1* (*PHOT1*), and *phytochrome1* (*PHYB*) [132,133]. Auxin-mediated light signals not only participate in phototropic responses, but also in plant shade avoidance syndrome (SAS) [26]. Shade-tolerant plants, such as *Arabidopsis thaliana*, need a certain degree of shade to grow normally [28]. SAS is mainly reflected in the morphological changes in plants, such as stem and petiole phototropic bending, delayed leaf development, and downward growth [27]. Changing the ratio of red to far-red light (R:FR) can effectively simulate plant shade responses, and therefore is widely used in shade response research [29,30]. In natural environments (high R:FR), the red light receptor phytochromeB (PHYB) inhibits plant phototropic responses; while under shading conditions, PHYB promotes plant phototropism by regulating the transcriptional activity of three important *basic Helix-Loop-Helix* (*bHLH*) transcription factors phytochrome interacting factors (PIFs) (PIF4, PIF5, and PIF7) [31]. The latest study showed that *SAV4* (*Shade Avoidance 4*) participates in the shade response of plant hypocotyls by regulating ABCB1-mediated auxin polar transport [24].

## 7. Auxin Signaling Pathway

**Figure 4:**
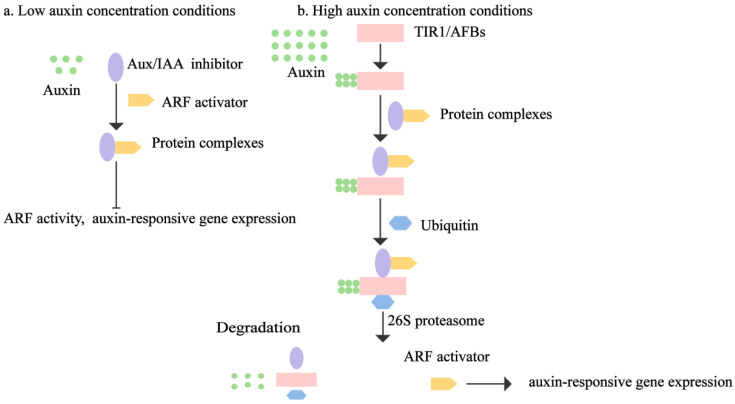
The auxin signaling transduction pathway in plants. Under low auxin concentration conditions, the auxin transduction repressor auxin/indole-acetic acid protein (Aux/IAA) forms a heterodimer with the auxin response factor (ARF), which inhibits the transcriptional activity of ARF, resulting in the suppression of auxin response gene expression. Under high auxin concentration, the auxin receptor transport inhibitor response 1 (TIR1) binds to Aux/IAA, ubiquitinates and degrades AUX/IAA by the action of the 26S proteasome, and ARF is released, activating the expression of auxin-responsive genes.

The auxin signaling pathway consists of auxin/IAA transcriptional inhibitors, auxin response factors (ARFs), and receptor proteins transport inhibitor response 1 (TIR1)/auxin-signaling F-box (AFB) (Figure 4) [134]. When auxin concentrations are low, Aux/IAA inhibitors bind to the ARF transcription factor, thereby inhibiting ARF activity. The binding of auxin to receptor TIR1/AFBs allows TIR1 to easily bind Aux/IAA proteins and induces a ubiquitination reaction [135]. Following degradation by the 26S proteasome, the AUX1/IAAs protein complex releases ARF, thereby initiating the regulation of downstream genes [136]. Research has shown that four Aux/IAA-ARF combinations, known as auxin signaling modules, are related to different stages of lateral root growth [137].

Signal transduction is an important link in plant auxin research, and there are four main auxin signal transduction pathways: The TIR1/AFB-Aux/IAA-TPL-ARFs pathway, the TMK1-IAA32/34-ARFs pathway, the TMK1/ABP1-ROP2/6-PINs or RICs pathway, and the SKP2-AE2FC/DPB pathway [138]. TIR1/AFB-Aux/IAA-TPL-ARFs is an extensively researched and widely recognized signaling pathway originating from the nucleus [139]. The first two pathways mediate the expression of auxin downstream genes by regulating ARF transcription factors, while the last two pathways directly activate some auxin efflux proteins and mediate the fast non-genomic effects induced by auxin [140]. It has been demonstrated that TIR1/AFB growth hormone signaling possesses a non-transcriptional branch that regulates rapid cellular processes, including cytoplasmic Ca^2+^ spiking and membrane depolarization. These processes have been associated with root growth inhibition [141]. In land plants, the TIR1/AFB receptor has adenylate cyclase (AC) activity, which contributes to root growth regulation by TIR1/AFB signaling and produces cAMP as a second messenger in this process. But this process still requires the involvement of an unknown mechanism [142]. Transmembrane kinase (TMK) has been demonstrated to mediate both transcriptional and non-transcriptional auxin signaling in Arabidopsis. Furthermore, it has been shown to activate Rho GTPase, which in turn controls the cytoskeleton [52,143]. Different TMKs have different roles in auxin signaling. Accumulation of growth hormone on the concave side of the apical hook stimulates TMK1 cleavage, which in turn leads to cytoplasmic and nuclear translocation cations that regulate gene transcription by stabilizing two nonclassical Aux/IAA proteins [144]. TMK4 has been demonstrated to regulate BR-mediated plant development [145] and to be involved in the negative regulation of growth hormone biosynthesis [52]. It has been shown that TMK1 inhibits plant growth by regulating ABI1/2, which mediates ABA signaling enhanced by high concentrations of auxin. Thus, TMKs coordinate growth hormone signaling with other signaling cascades, and TMKs may mediate differential growth hormone responses by phosphorylating different downstream components [146]. S-Phase Kinase-Associated Protein 2A (SKP2A) is a cell cycle-regulated F-box protein that controls the stability of at least two cell division transcriptional factors, E2FC and DPB [147]. Previous study has showed that auxin can regulate cell division through the SKP2A pathway. In the presence of auxin, SKP2A promotes degradation of cell cycle targets; additionally auxin enhances SKP2A protein hydrolysis to impede its excessive functionality [148]. Overexpression of SKP2A results in increased cell division and induces lateral root primordia (LRP) formation, a process known to be dependent on auxin signaling [149]. Although the SKP2A-E2FC/DPB pathway has been proposed, we lack evidence of many of its parts, thus more research is required. For example, SKP2A binding to SCF is able to target degradation of downstream E2FC and DPB; is it regulated by the proteasome in the same way, and what are the effects of degradation on the plant? Why does the mutation SKP2A not have a significant effect on plant growth and development?

## 8. Interaction of Auxin with Other Hormones

The signaling pathways of various hormones in plants often cross each other, forming a complex regulatory network. To date, the most attention has been paid to the interaction between auxin and cytokinin (CTK), jasmonic acid (JA), and abscisic acid (ABA).

### 8.1. Interaction between Auxin and CTK

The interaction between auxin and CTK involves both antagonistic and synergistic effects. In Arabidopsis, CTK signaling was shown to regulate the rate of auxin (IAA-indole-3-acetic acid) biosynthesis [150]. CTK regulated the auxin gradient to control the growth of lateral roots [151]. Auxin might modulate the mutual binding of CTK molecules and inactivate CTK. For example, auxin regulates CTK levels in the stem by inducing the expression of *Cytokinin oxidase/dehydrogenase* (CKX), suppressing the expression of *ATP/ADP isopentenyltransferase* (IPTs), and promoting the expression of strigolactone biosynthesis-related genes [152,153]. Auxin might modulate the mutual binding of *CTK* molecules and inactivate CTK [154]. Moreover, Nordstrom found that auxins inhibit CTK biosynthesis mainly through the *isopentenyladenosine-5’-monophosphate (iPMP)-independent* pathway and that this negative regulation is a fast-acting process [155]. During root development, antagonistic effects were observed, with auxin encouraging adventitious root production and exogenously applied physiological CTK inhibiting root formation and reversing IAA’s effects [156]. Moreover, the rice auxin response factor OsARF25 can bind to the promoter of the cytokinin oxidase gene *OsCKX4* and activate its expression, thereby enhancing CTK metabolism [157].

### 8.2. Interaction between Auxin and JA

Numerous investigations have found that JA is involved in PAT and biosynthesis. For example, Li discovered that JA not only regulates auxin production by stimulating *ASA1* expression, but it also influences PAT [158]. Furthermore, JA can stimulate auxin production genes *ASA1* and *YUC2* by increasing the expression of the transcription factor *ERF109*, thus enhancing auxin biosynthesis [159]. Furthermore, both JA and IAA regulate transcription factor *WRKY57*, which can modulate JA and IAA signaling pathways in feedback [160].

### 8.3. Interaction between Auxin and ABA

It has been shown that there are both antagonistic and synergistic effects between them. As reported previously, PYL8 mediates the synergistic action of ABA and auxin to promote lateral root growth after sprouting [161]. WRKY46 contributes to the forward inhibition of osmotic/salt stress-induced LR inhibition by regulating the ABA pathway and growth hormone homeostasis [162]. During seed dormancy, auxin can stimulate *ABI3* expression by activating ARF10/16, thereby activating the ABA signaling pathway [163]. Additionally, ABA can modulate auxin signaling, and the ABA receptor PYL8 activates MYB77, which increases the production of auxin response genes [161].

### 8.4. Interaction between Auxin and Ethylene

Ethylene can regulate the synthesis of auxin. Exogenous 1-aminocyclopropane-1-carboxylic acid (ACC) treatment increased the expression of *AUX1*, *PIN3*, and *PIN7* while inhibiting lateral root development [118,164]. Auxin also regulates the production of ethylene. Upon *SlARF2* silencing, tomatoes produced less ethylene and expressed fewer ripening-related genes such as *RIN*, *CNR*, *NOR*, and *TAGL1* [117]. Exogenous auxin treatment of peaches resulted in increased *PpACS1* expression and ethylene production [165,166]. The expression of *MdARF5* was promoted in apples treated with NAA, which combined with the promoters of *MdERF2*, *MdACS3a*, *MdACS1*, and *MdACO1* to induce ethylene biosynthesis [167]. Moreover, *CpARF2* interacts with *CpEIL1* in papaya to promote *CpACS1* and *CpACO1* transcription and regulate fruit ripening [168].

In addition, auxin interacts with other hormones such as gibberellin, brassinosteroids, and salicylic acid. For example, in rice, auxin and gibberellin can regulate the negative gravity response of rice stem by antagonizing the expression of *XET* [169]. During hypocotyl growth, brassinosteroids can activate the auxin signaling pathway by inducing the transcription of *IAA19* and *ARF7* by BRASSINAZOLE-RESISTANT 1 (BZR1) [170]. Salicylic acid can inhibit the auxin biosynthesis induced by H_2_O_2_ by inhibiting the function of CATALASE2 [171].

## 9. The Role of Auxin in Stress

### 9.1. Heat Stress 

Auxin plays an important role in heat stress-induced thermal morphogenesis, including stem (hypocotyl) elongation and subleaf glands [172]. Plants respond to high-temperature stress through auxin anabolism, polar transport, and signal transduction. High heat increases the level of free IAA by triggering the dominant two-stage IAA biosynthesis pathway from Trp to 3-IPA via TAA-1, then oxidative decarboxylation of 3-IPA to IAA catalyzed by flavin monooxygenases of the YUC subfamily [57,173,174]. In *Arabidopsis*, the homeostasis, turnover, and distribution of free IAA in the hypocotyl are controlled by the IAA amidosynthetase VAS2-GH3.17 under high temperatures [175]. In the *Arabidopsis* root system, the heat-stimulating protein HSP90 acts as a molecular chaperone for the auxin receptor TIR1 and influences the polar distribution of the auxin transporter protein PIN1 in the plasma membrane, thereby creating a concentration gradient of auxin and regulating the plant root system, growth, and development [176]. In *Sorghum bicolor* L., high temperatures upregulated the expression levels of most *SbARF* genes, and the *SbARF17/24* genes were found to be heavily expressed and accumulated in vascular tissue [177]. The above results indicated that auxin plays an important role in plant resistance to high-temperature stress and thermal stimulus transduction.

### 9.2. Flood Stress 

Flooding prevents oxygen from reaching the roots, inhibits adventitious root (AR) formation, and might lead to moderate to severe root damage [178,179]. The formation of ARs is largely dependent on local auxin biosynthesis and translocation. Qi et al. (2023) found that endogenous auxin levels in hypocotyls increased, while externally applied NAA enhanced AR formation, at 72 h after flooding [180]. In addition, auxin treatment upregulated the expression levels of ethylene biosynthesis genes (*CsACS1*, *CsACS2*, *CsACO5*) and ROS signaling genes (e.g., *CsRBOHB* and *CsRBOHF3*) under flooding stress [181]. Gao et al. (2022, 2023) found that *Aux/IAA* gene expression and the auxin content were downregulated after 8 days of waterlogging, while exogenous spermidine alleviated waterlogging stress in roots and increased the auxin content in *Phyllostachys praecox* [182,183,184]. These results indicated that auxins play an important role in coping with flooding stress in plants.

### 9.3. Cold Stress

Cold temperatures limit plant growth mainly by causing cold damage to tissues. Zhu et al. (2015) discovered that low temperatures reduced the expression of *PIN1/3/7* and auxin biosynthesis-related genes and decreased auxin accumulation, which in turn inhibited the division potential of *Arabidopsis* meristematic tissue cells [34]. In the auxin degradation pathway, relative expression levels of *Gretchen Hagen 3* gene (*GH3.3* and *GH3.6*) was upregulated by cold stress in *Cicer arietinum* shoots [185]. Moreover, overexpression of *CsARF5* enhanced cold stress tolerance in cucumber [186]. The cold stress significantly altered transcript levels of *SlSAURs* genes in Solanaceae species [187]. The levels of certain auxin response factors (*ARFs*; *TaARF8*,*TaARF9* and *TaARF21*) are reduced at low temperatures [188]. The changes in the expression of these genes suggest that low temperatures altered the expression of genes involved in auxin metabolism, thereby affecting auxin levels and inhibiting plant growth.

### 9.4. Salt Stress 

Excess salt disrupts plant physiological, biochemical, and molecular processes and salt stress is the second most important abiotic factor affecting global agricultural productivity [189]. Auxin improved salt tolerance in cucumber seedlings, and transcriptomic analysis revealed that auxin signaling genes *SAUR*, *Aux/IAA*, and *GH3* were downregulated in salt stress [190]. The expression of 5NG4-like, a key molecular transporter gene induced by auxin, was upregulated in seedlings treated with NaCl with exogenously added silicon [191]. It was hypothesized that auxin signaling genes play a key role in silica-mediated salt tolerance. However, functional studies are required to determine the underlying mechanisms.

### 9.5. Drought Stress 

Drought stress downregulated auxin genes in the auxin sub-pathway, including genes encoding auxin influx proteins, auxin response proteins (AUX/IAA), ARF, and GH3. RNA-Seq-based transcriptome analysis showed that melatonin upregulated 23 genes involved in growth hormone signaling, including *AUX/IAA*, *ARF,* and *SAUR*, in *Davidia involucrate* [192]. In the present study, overexpression of *OsIAA6* and *IbARF5* improved drought tolerance in rice and Arabidopsis, respectively [193,194]. In drought- and CO_2_-treated cucumber roots, IAA levels were decreased. By contrast, gibberellin (GA) had a significant inducing effect. Thus, auxin might regulate the response of cucumber to drought stress downstream of GA [181].

### 9.6. Heavy Metal Stress

Heavy metals damage plant cells by disrupting a variety of physiological processes [195]. Cadmium (Cd) affects auxin biosynthesis and transport, thereby altering the formation of quiescent centers (QC), whereas exogenous auxin restores normal root development [196]. Cd increased the expression of IAA influx carrier *AUX1* and strongly repressed the expression of *PIN5*, and *OsPIN5b* was involved in the regulation of IAA homeostasis, transport, and distribution [197,198]. In contrast, arsenic (As) reduced the expression of the *AUX1* and the efflux carrier *PIN5*. However, both Cd and As affect adventitious root (AR) and lateral root (LR) development through the regulation of auxin carriers in turn [198,199]. A recent study highlighted the role of auxin in the response of cucumbers to cadmium stress. The study showed that exogenous application of selenium significantly inhibited the harmful effects of cadmium. Auxin binding protein (ABP19a-like) levels were higher in Se-treated seedlings than in cadmium-treated seedlings [200]. However, further functional studies are needed to validate auxin’s involvement cadmium mitigation or other heavy metal stresses.

## 10. Conclusions and Perspectives

In recent years, great progress has been made in understanding the mechanisms of auxin sensing and signaling. Moreover, the interaction between auxin and environmental signals in controlling plant growth and development has garnered increased interest. Many studies have shown that environmental signals, particularly abiotic stress, directly regulate some key genes of auxin synthesis and metabolism, polar transport, and signaling. However, the molecular mechanism of auxin regulation under abiotic stress requires further investigation. Based on the current research status, we suggest that future research directions should concentrate on the following areas. Firstly, in the face of the increasingly serious problem of global warming, the possible role of temperature signaling and its molecular mechanisms require further study. For example, in male sterility of plants under high-temperature stress, in addition to changes in the endogenous auxin content, more evidence is required to support which transcription factors are directly involved in the effects of high-temperature stress on plant growth and development. Spatial and temporal regulation of auxin synthesis is critical for plant development. It combines gene editing technology to precisely regulate the expression of key enzymes for auxin synthesis, thus realizing precise control of plant growth. A recent study has shown that ultra-rapid global phosphorylation downstream of auxin sensing on ABP1-TMK cell surfaces allows auxin responses to be completed within seconds. Notably, previously, TIR1/AFB-mediated slow auxin signaling responses tended to be in the 20–30 min. In summary, the auxin fast response may play a pivotal role in plant stress as well as in signaling cascade responses [201]. It deserves to be explored further. 

Additionally, current research on Aux/IAA in the auxin signaling pathway has focused on plant growth and development, but little attention has been paid to the role of Aux/IAA in the auxin-mediated response to environmental interactions (e.g., drought, heavy metals, nutrient deficiencies, and other abiotic stresses) and it is critical to understand how auxin interacts with other hormones in this process. Future genetic investigations, together with computational modeling, will enable the identification of novel candidate genes that modulate Aux/IAA, and hence the overall auxin signaling regulation network. Moreover, Wang et al. (2004) [202] found that strigolactones (SLs) reduced the inhibitory effect of *WRKY41* on the expression of CBF/*dehydration response element binding factor 1* (*DREB1)* to promote cold tolerance in plants. It is because the effects of SLs and auxin on *wrky* genes in plants under stress, but the specific mechanism is still unclear. In synergism or antagonism with other hormones and signaling molecules, auxin may affect photosynthesis, plant antioxidants, arbuscular mycorrhizal (AM) symbiosis, etc., thereby mitigating the damage caused by different abiotic stresses on plants. This deserves to be further explored to provide new ideas and approaches for agriculture and biotechnology applications. 

## Data Availability

The raw data supporting the conclusions of this article will be made available by the authors on request.
